# The efficiency of low-dose hepatitis B immunoglobulin plus nucleos(t)ide analogs in preventing posttransplant hepatitis B virus recurrence

**DOI:** 10.3906/sag-1808-86

**Published:** 2019-08-08

**Authors:** Sezgin VATANSEVER, Rasim FARAJOV, Hüseyin Cahit YILMAZ, Murat ZEYTUNLU, Murat KILIÇ

**Affiliations:** 1 Department of Gastroenterology, Atatürk Training and Research Hospital, İzmir Katip Çelebi University, İzmir Turkey; 2 Department of Liver Transplantation, Kent Hospital, İzmir Turkey

**Keywords:** Liver transplantation, Hepatitis B immunoglobulin, dose, recurrence

## Abstract

**Background/aim:**

In this study, the efficiency of using low-dose hepatitis B immunoglobulin (HBIG) plus antiviral treatment according to individual needs has been evaluated in posttransplant hepatitis B virus (HBV) patients.

**Materials and methods:**

We retrospectively evaluated 179 patients who were admitted between 2009 and 2014. Five thousand IU intravenous HBIG was given in the anhepatic phase, and 400 IU/day intramuscular (IM) HBIG was given in the posttransplant period. After HBsAg seroconversion, 400 IU IM HBIG was continued as prophylaxis every two weeks.

**Results:**

The average follow-up period was 26 (2–65) months. Seventy patients had hepatocellular carcinoma (HCC). The HBV recurrence was 4.5% in the first year, and 5.8% in the third year. The HBsAg became negative in 11 (2–63) days, and anti-HBs became positive in 9 (1–31) days. HBsAg positivity occurred in 6 patients during the follow-up period. Five of these patients were those who underwent transplantation due to HCC. In 5 of the HCC patients, in whom HBsAg became positive, tumor recurrence was observed after 0.3–9.9 months. HBsAg positivity was more frequently detected in patients with HCC (P = 0.009).

**Conclusion:**

The HBV recurrence should be evaluated as a predictor of the HCC recurrence in patients who were transplanted due to HCC.

## 1. Introduction

Prophylactic hepatitis B immunoglobulin (HBIG) in combination with oral antiviral therapy has become a standard treatment for patients who undergo liver transplantation surgery due to Hepatitis B [1–3]. One of the most important factors affecting posttransplant costs is the amount of consumed HBIG [1–3]. HBIG reduces the number of virions within the circulation and ensures the lysis of infected hepatocytes. However, escape and surface mutants may sometimes develop in the course of treatment. Oral antivirals, on the other hand, inhibit the reinfection of hepatocytes as well as the replication of the mutant virions that have developed. Thus, the combination therapy generates a synergistic effect [4]. There are HBIG regimens used in different ways [5–9] as follows; 1) In anhepatic phase, HBIG is administered intravenously (IV) as 4000–10,000 IU/mL [10]. 2) For the initial 3–7 days after the liver transplantation, 2000 to 10,000 IU HBIG a day is administered in an intramuscular or intravenous way. 3) In order to ensure the maintenance of anti-HBs > 100 IU/L after the transplantation, either a high-dosage medication is administered as 10,000 IU of HBIG with a 4 week-cycle or a low-dosage medication is administered as 400–2000 IU of HBIG with a 2-week cycle [11,12].

In various studies, it was seen that HBIG used in low doses could be as effective as high doses. In different studies, it was shown that 1- to 2-year HBV recurrence for HBIG 400–800 IU with a maintenance dose applied intramuscularly every two weeks proved to be below 10% [13–15].

In this study, we aimed to investigate the efficiency of low-dose HBIG plus nucleos(t)ide analogs and the factors affecting hepatitis B recurrence in patients who underwent liver transplantation due to hepatitis B.

## 2. Materials and methods

We retrospectively evaluated a cohort of 179 (154 M/25 F) patients from the liver transplantation center who underwent liver transplantation due to Hepatitis B-related decompensated cirrhosis and hepatocellular carcinoma (HCC) between July 2009 and February 2014. The mean age of the patients was 51.8 ± 9.6 (15–71) years.

Research involving human subjects (including human material or human data) that is reported in the manuscript is in compliance with the Helsinki Declaration. This study was approved by the Ethical Committee. 

### 2.1. Immunosuppressive regimen

 A total of 500 mg methylprednisolone was administered on the day of operation. Starting from the first postoperative day, a daily dose of 100 mg was administered. The dose was reduced by 10 mg each day until it reached a minimum daily dose of 20 mg. This dose of 20 mg/day was given for a month, and then was decreased by 5 mg every month. After 4–6 months, prednisolone was discontinued. On the first postoperative day, 1 mg tacrolimus 2 × 1 po was administered, and the dose was augmented to maintain a blood level of 5–15 ng/mL; 15 ng/mL to begin with and 5 ng/mL to reach at the end of the first year and afterwards.

On the other hand, sirolimus or everolimus was started in the patients who had an increase of creatinine level or in whom specific side effects developed against tacrolimus. Mycophenolate mofetil (MMF) was also added for the patients for whom immunosuppressive therapy remained insufficient. Each patient was given oral antiviral treatment (Lamivudine (LAM), Adefovir (ADV), Entecavir (ENT), Tenofovir (TDF), Telbivudine (LdT)) in the pretransplant period. HBV-DNA levels became negative after oral antiviral treatment prior to transplantation in all of the patients. The preoperative oral antiviral treatment was also continued postoperatively for all patients.

### 2.2. HBV reactivation prevention strategy

 The patients were administered 5000 IU IV HBIG in the anhepatic phase. During the posttransplant period, HBIG was continued at 400 IU IM daily. HBsAg and anti-HBs status was examined 3 days a week (Monday, Wednesday, and Friday). After HBsAg became negative (HBsAg < 1.0 S/CO (Negative)) and anti-HBs (anti-HBs > 10 IU/L (positive)) became positive, the daily HBIG administration was changed to HBIG 400 IU IM once every 2 weeks. HBsAg status was studied through the use of ARCHITECT HBsAg Qualitative II device. After HBsAg became negative, it was followed up at the intervals of 3–6 months. The HBV recurrence was accepted as the positivity of HBsAg with/without detectable serum HBV-DNA.

Anti-hepatitis C virus (HCV) positivity was confirmed by HCV-RNA using real-time PCR.

### 2.3. Statistical analysis

In the analysis of the relevant data, the SPSS 17.0 Statistics Program (SPSS Inc., Chicago, IL) was used. All of the digital values are given as mean value ± standard deviation or in the median form. The chi-square test was used for the categorical variables. In addition, the normality of the groups, as well as their compliance with homogeneity, was evaluated. The data incompatible with the normal distribution were evaluated using the Mann–Whitney U test, whereas those compatible with the normal distribution were evaluated using Student’s t-test. The Kaplan–Meier method was used to estimate the survival curves. The survival curves were compared across the ordered categories with the Log-Rank test for trend. The relationship between the dependent and independent variables was investigated through regression correlation analysis. P < 0.05 was accepted statistically significant.

## 3. Results

The demographic data and the characteristics of the study group are shown in Table 1. The mean follow-up period was 26 (2–65) months. The HBsAg measurements of all the patients were positive at the time of the transplantation operation. The mean HBIG used by the patients was 20,885 ± 2898 IU in the first year, 30,718 ± 2366 IU in the second year, and 42,362 ± 3028 IU in the third year. With the use of our HBIG treatment regimen, the anti-HBs titration in 17 (9.5%) patients did not exceed 100 IU/L.

**Table 1:** Characteristics of the patients.

	Total n = 179	HCC n = 70	non-HCC n = 109	P-value*
Age	51.8 ± 9.6	56.2 ± 7.7	48.8 ± 9.6	<0.001
Sex (F/M)	25/154	5/65	20/89	0.046
Follow-up period (months)	26 (2–65)	20 (2–63)	27 (2–65)	0.016
MELD	14 (6–35)	10 (6–29)	16 (6–35)	<0.001
CHILD	8 (5–15)	7 (5–13)	9 (5–15)	<0.001
Weight of patients (kg)	78 ± 14	78 ± 15	77 ± 13	0.267
Weight of graft (gr)	788 ± 151	760 ± 147	803 ± 151	0.097
Weight of explant liver (gr)	1180 ± 320	1273 ± 340	1115 ± 292	0.001
Cadaver/living donor	33/146	16/54	17/92	0.241
Log HBV-DNA before treatment	5.3 ± 1.6	5.4 ± 1.5	5.0 ± 1.6	0.233
HBeAg	15 (8.4%)	7 (10%)	8 (7.3%)	0.725
Anti-Delta	56 (31.2%)	11 (15.7%)	45 (41.3%)	0.001
HBs Ag negativity (days)	11 (2–63)	10 (2–63)	11 (2–41)	0.777
Anti-HBs positivity (days)	9 (1–31)	8.5 (2–24)	9 (1–31)	0.982
Anti-HBs > 100 IU/ml (days)	10 (2–60)	10 (2–32)	11 (2–60)	0.546
Antiviral (TDF/ENT)	63 (35.2%)	24 (34.3%)	39 (35.8%)	0.879
Antiviral (LAM/ADV/LdT)	116 (64.8%)	46 (65.7%)	70 (64.2%)	0.901

*Comparison of the patients with HCC and non-HCC.

HBsAg recurrence occurred in 6 patients within 16.1 months (Table 2). The HBV recurrence was determined to be 4.5% in the first year, 5.8% in the second year, and 5.8% in the third year.

**Table 2:** The characteristics of the patients in whom HBV recurrence has occurred.

Age	Sex	Etiology	Antiviraldrug	HCCexistence	HBV recurrence (months)	HCC recurrence (months)	Outcome (months)
55	Male	HBV-HDV	LAM	Yes	3.5	10.1	14 (died)
61	Male	HBV	LAM	Yes	5	13	15 (died)
52	Male	HBV	ENT	Yes	6.1	6.4	7.3 (died)
46	Male	HBV-HDV	LAM	No	10.4	-	64 (alive)
62	Male	HBV	ADE+LAM	Yes	10.5	20	34 (died)
62	Male	HBV	TEN	Yes	16.1	26	45 (died)

In one patient in whom anti-HBs titration did not exceed 100 IU/L, HBV recurrence occurred. This patient had no diagnosis of HCC. 

Thirty-three of the transplant patients (18.4%) received an organ from a cadaver whereas 146 patients received an organ from a living donor. HCC was diagnosed in 70 of the patients. Of these patients, 15 were incidentally detected in the explant liver. Thirty of the patients (42.8%) with HCC were those that had exceeded the Milan Criteria (a single tumor ≤5 cm or a maximum of 3 total tumors with none >3 cm).  Four patients (2.2%) had HCV coinfection and 56 patients (35.2%) had HDV coinfection. HCV-RNA levels varied between 104 and 106 IU/mL in the patients with HCV. HBV recurrence was not observed in these patients.

Of 6 patients with HBV recurrence, 5 had been transplanted due to HCC. During the follow-up period, 14 patients developed HCC recurrence and 5 (35.7%) of them had HBsAg recurrence before HCC recurrence. Thirteen of these patients who developed HCC recurrence exceeded the Milan Criteria. In addition, all the patients who had HBsAg recurrence exceeded the Milan Criteria. Prominent HBsAg positivity was detected more frequently in patients with HCC than without HCC (P = 0.009) (Figure). With the Cox regression multivariate analysis, HBV recurrence was determined to be associated with the presence of HCC prior to LT (OR = 10.17; 95% CI: 1.18–87.14; P = 0.034). HBV recurrence occurred in 2 patients with HDV hepatitis. The HDV was not effective on HBV recurrence in liver transplanted patients (OR = 0.406; 95% CI: 0.42–3.90; P = 0.435).

**Figure:**
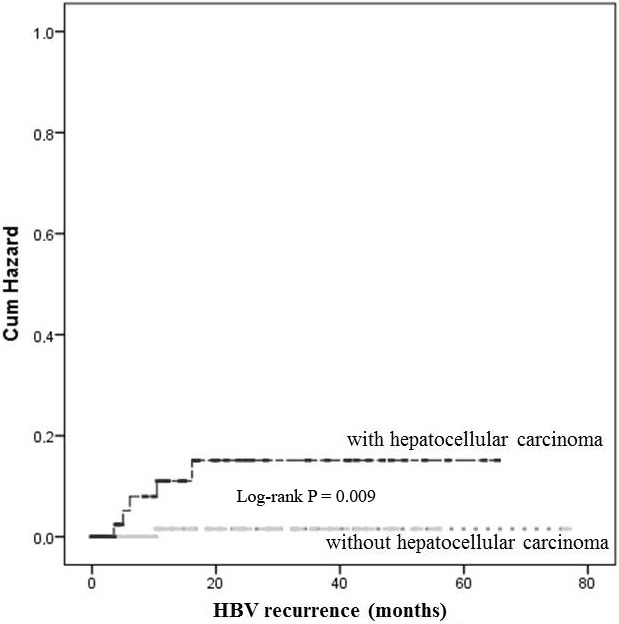
HBV recurrence of the patients with HCC and without HCC.

None of the patients died because of liver failure due to HBV recurrence. An elevation of liver enzyme due to recurrence occurred in 1 patient. The antiviral treatment of this patient was replaced with the potent antiviral agent (TDF).

Sixty-three patients were given a potent antiviral agent. HBV recurrence developed in 2 of these patients. There was no significant difference between patients who were given potent (TDF/ENT) and nonpotent antiviral agent (OR = 0.962; 95% CI: 0.16–5.76; P = 0.967).

No relationship was determined among patients’ weight along with the HBsAg negativity (P = 0.221, r = 0.056), anti-HBs positivity (P = 0.599, r = 0.013), and the anti-HBs that were >100 IU/L (P = 0.708, r = –0.042).

## 4. Discussion

Hepatitis B is still the most common cause of liver transplantation in developing countries [16]. Although different treatments and HBIG practices have been developed throughout the years, there is still no standard treatment protocol to prevent postoperative recurrence. The HBIG applied during our study period was seen to have ensured HBsAg negativity as much as the other regimens [13–15]. The total HBIG dose used in our study is lower than the low dose of HBIG applied in other previous studies [3,17–19].

In a study conducted by Jiang et al., which comprised 233 patients and also included an average 31-month follow-up period, an average of 23,000 IU HBIG was used in the first year, whereas in the second year, 34,000 IU HBIG was used. Recurrence was identified in 14 (6.2%) patients [20].

On the other hand, in another low-dose protocol referred to as the Australasian Protocol, <10% recurrence took place in those who used 15,000 IU HBIG in the first year and those who used 25,000 IU HBIG in the second year [8,21].

In another study in which 114 patients received a low-dose HBIG, 2000 IU HBIG was used in the anhepatic phase, 800 IU HBIG was used monthly in maintenance, and thus, a cumulative of 18,000 IU HBIG was used in the first year. During the 20-month follow-up period, recurrence was seen in 16 patients. The recurrence was determined to be as high as 13.5% in the first year and 15.2% in the second year [22]. In addition to that, the rate of HBV recurrence was reported to be 1%, 3%, and 3% at 1, 3, and 5 years respectively with low-dose of HBIG plus antiviral treatment in our region and HCC recurrence was reported as a risk factor for HBV recurrence (HR: 12.3, P = 0.02) [23].

In our study, we determined that HBV recurrence was lower than that in other studies that utilized a low-dose HBIG regimen. This finding may be due to lower HBeAg positivity, lower HBV-DNA levels, higher HDV co-infection rate, and more frequent use of potent antivirals in the pretransplantation period in our patients [15,24–27]. Another factor was the unlimited administration of HBIG until HBsAg became negative, not depending on a given period specified by the protocol.

In our study, while the day that HBsAg became negative was 11 (2–63), the anti-HBs positivity was observed on day 9 (1–31). The time when anti-HBs reached above 100 IU/L corresponds to the same period when HBsAg became negative, such as 10 (2–60) days. As is also recommended in several studies, the goal to raise anti-HBs above 100 IU/L ensures long-term HBsAg negativity [28]. It was observed that 90.5% of anti-HBs titration reached above 100 IU/L. No HBsAg positivity occurred in any of the patients whose anti-HBs were over 100 IU/mL and who had no HCC.

HBsAg reactivation occurred only in 1 (0.9%) of the patients without HCC. This patient’s anti-HBs did not rise above 100 IU/L. In a study where a similar amount of HBIG was used yearly in patients without HCC, HBV recurrence occurred at a rate of 2.3% in the first year and 6.2% in the third year [20].

The most striking finding in our study is the fact that HBV recurrence is higher in the patients with HCC (8.1% and 0.9%) (P = 0.009). However, in our study, the recurrence in both of the groups was lower when compared with the former studies.

On the other hand, in a study conducted by Kıyıcı et al., HBV recurrence in patients with HCC during the 31-month follow-up period was determined to be higher in comparison to those without HCC (23.6% and 5.5%, respectively) [18]. Similarly, in a study conducted by Faria et al., HBV recurrence was higher in those with HCC during the 15-month follow-up period (35.5% and 4.4%) (P < 0.0001) [29].

Faria et al. showed that there was cccDNA in both HCC cells and in nontumor cells in the explanted livers from the patients who had recurrent HCC, which suggested that the HBV replication might also appear in tumor cells [29]. Since the proliferation ability of immortal tumor cells is rather high, the replication is also high, indicated by the presence of HBsAg within the blood [30,31]. Thus, in this study, it was observed that HCC recurrence appeared 0.3–9.9 months after HBsAg became positive and HBV recurrence should be evaluated as a predictor of HCC recurrence.

The primary restriction of our study is that by lowering the dose of HBIG more and more, the optimum amount of HBIG usage could not be investigated. In addition, the antiviral treatment was not homogeneous and we did not study HBV-DNA polymerase gene mutation in patients with HBV recurrence. 

In conclusion, low-dose HBIG plus antiviral treatment is effective in prevention of HBV recurrence after liver transplantation and HBV recurrence is a predictor of HCC recurrence after liver transplantation.
